# Use of Tox21 Screening Data to Evaluate the COVID-19 Drug Candidates for Their Potential Toxic Effects and Related Pathways

**DOI:** 10.3389/fphar.2022.935399

**Published:** 2022-07-14

**Authors:** Srilatha Sakamuru, Ruili Huang, Menghang Xia

**Affiliations:** Division of Pre-clinical Innovation, National Center for Advancing Translational Sciences (NCATS), National Institutes of Health (NIH), Bethesda, MD, United States

**Keywords:** COVID-19, coronavirus, high throughput screening, *in vitro* assay, drugs

## Abstract

Currently, various potential therapeutic agents for coronavirus disease-2019 (COVID-19), a global pandemic caused by the severe acute respiratory syndrome coronavirus 2 (SARS-CoV-2), are being investigated worldwide mainly through the drug repurposing approach. Several anti-viral, anti-bacterial, anti-malarial, and anti-inflammatory drugs were employed in randomized trials and observational studies for developing new therapeutics for COVID-19. Although an increasing number of repurposed drugs have shown anti-SARS-CoV-2 activities *in vitro*, so far only remdesivir has been approved by the US FDA to treat COVID-19, and several other drugs approved for Emergency Use Authorization, including sotrovimab, tocilizumab, baricitinib, paxlovid, molnupiravir, and other potential strategies to develop safe and effective therapeutics for SARS-CoV-2 infection are still underway. Many drugs employed as anti-viral may exert unwanted side effects (i.e., toxicity) via unknown mechanisms. To quickly assess these drugs for their potential toxicological effects and mechanisms, we used the Tox21 *in vitro* assay datasets generated from screening ∼10,000 compounds consisting of approved drugs and environmental chemicals against multiple cellular targets and pathways. Here we summarize the toxicological profiles of small molecule drugs that are currently under clinical trials for the treatment of COVID-19 based on their *in vitro* activities against various targets and cellular signaling pathways.

## Introduction

The outbreak of coronavirus disease-19 (COVID-19), a global pandemic caused by a novel coronavirus designated as SARS-CoV-2 (Severe Acute Respiratory Syndrome Coronavirus 2), has led to more than 5.2 million deaths and 261 million infected patients worldwide up to the end of 2021. To date, there is no effective treatment for COVID-19, and the search for an effective treatment has relied mainly on the repurposing of existing drugs, for which the long approval process could be shortened with established safety profiles and dosing regimen. So far, the U.S. Food and Drug Administration (FDA) has approved remdesivir (antiviral drug) to treat the COVID-19 patients (12 years of age and older) requiring hospitalization (https://www.fda.gov/news-events/press-announcements/fda-approves-first-treatment-covid-19). Remdesivir has been shown to speed up the recovery time in COVID-19 patients with lower respiratory tract infection in a randomized, placebo-controlled trial (Beigel et al., 2020). However, the mortality rates still persist with the use of remdesivir and other strategies were evaluated including the use of the antiviral drug in combination with a Janus kinase (JAK) inhibitor, baricitinib, but this combination was shown to be associated with adverse effects (Kalil et al., 2021). The NIH COVID-19 treatment guidelines recommended the use of dexamethasone, an anti-inflammatory corticosteroid, in patients with severe COVID-19, as the patients with severe disease conditions can develop an inflammatory response that leads to pulmonary injury and dysfunction. In a Randomized Evaluation of COVID-19 therapy trial, dexamethasone was given to critically ill patients in the United Kingdom and was found to lower 28-day mortality only among the patients who were on mechanical ventilators and in the patients requiring oxygen (Group et al., 2021). Since the pandemic started in early 2020, massive searches for new and effective therapies have been underway and drug repurposing remains a cost-effective approach in bringing the best available drug candidates in the shortest time to COVID-19 patients. The safety and effectiveness of the drugs for COVID-19 therapy were being evaluated in numerous clinical trials worldwide. An international clinical trial, called the RECOVERY trial (Randomized Evaluation of COVID-19 therapy), is investigating whether treatments with several antiviral, antimalarial, or antibacterial drugs can prevent death in COVID-19 patients. In this review, we will examine the drugs that are in current clinical and RECOVERY trials for COVID-19 patients.

SARS-CoV-2 is genetically related to the severe acute respiratory syndrome coronavirus (SARS-CoV) that emerged in 2002 (Rabaan et al., 2020). SARS-CoV-2 is believed to invade human body by binding its viral spike (S) protein to the host cell receptor, angiotensin-converting enzyme 2 (ACE2) and then the host cell serine protease, called transmembrane protease serine 2 (TMPRSS2), activates the S protein by proteolytic cleavage (Hoffmann et al., 2020). Upon viral infection of the host cell, various cellular signaling pathways are activated to facilitate viral replication leading to inflammation that could result in acute lung injury (Hemmat et al., 2021). SARS-CoV-2 infection leads to the recruitment of several cellular signaling pathways, such as inducing the apoptosis of lung cells by upregulating SMAD7 (Yeung et al., 2016), a protein involved in the transforming growth factor beta (TGFβ) signaling pathway, NF-kB activation that induces the inflammatory response against respiratory viruses (Canton et al., 2018), and upregulation of the transcription factor p53 that induces the expression of the genes necessary for apoptosis and inhibits viral replication (Munoz-Fontela et al., 2008). Drugs that can target several of these activated cellular signaling pathways could have a therapeutic value in developing a combination treatment strategy with other antiviral drugs. TMPRSS2, one of the main host cell factors that aid in SARS-CoV-2 pathogenicity and its transcription, is regulated by the androgen receptor and its ligands (Lin et al., 1999). Several other signaling pathways including activator protein 1 and autophagy could also be potential targets as their activity profiles were shown to correlate well with that of a cytopathic effect assay of SARS-CoV-2 (Zhu et al., 2021). To evaluate and characterize the potential toxicity profiles of some COVID-19 drug candidates, we used existing Tox21 (Toxicology in 21st century) screening datasets in this study. First, we reviewed a group of COVID-19 drug candidates (Table 1) that are present in the Tox21 chemical library, and then evaluated the *in vitro* activities of these drugs based on their toxicity profiles generated from quantitative high-throughput screening (qHTS). We included the drug candidates that are currently employed in the clinical trials of COVID-19 and the outcomes published from those treatment trials.

**TABLE 1:** Drugs employed in the current treatment trials of COVID-19.

Drug Candidates (CAS Registry Number)	Class	Mechanism of action/Target	COVID-19 treatment Trials	AC_50_ (µM)*
Artemisinin (63968–64–9)	Antimalarial	Parasite proteasome inhibitor (Bridgford et al., 2018)	Significantly shortened the time to reach undetectable SARS-CoV-2 in mild-to-moderate COVID-19 patients when given along with piperaquine (Li et al., 2021a)	2.32 ± 0.94 (ERα-bla-antagonist)
				2.44 ± 1.22 (ERRα-luc-antagonist)
				14.5 ± 10.8 (PGC/ERRα-luc-antagonist)
Azithromycin (83905–01–5)	Antibacterial	Protein synthesis inhibitor (Parnham et al., 2014)	No improvement in the clinical outcomes either given alone or in combination (Cavalcanti et al., 2020; Group, 2021)	3.41 ± 0.77 (Shh-luc-antagonist)
Bromhexine (611–75–6)	Mucolytic	TMPRSS2 inhibitor (Lucas et al., 2014)	Showed beneficial effect in COVID-19 patients with lung injury (Li et al., 2020)	4.60 ± 2.35 (AR-luc-antagonist)
				16.36 ± 8.90 (CAR-luc-agonist)
				14.05 ± 8.70 (ERα-bla-agonist)
				9.91 ± 1.78 (PR-bla-antagonist)
				24.34 ± 9.54 (PXR-luc-agonist)
Budesonide (51333–22–3)	Corticosteroid	GR activator (Kalola and Ambati, 2022)	Inhaled budesonide reduced time to recovery in COVID-19 patients with early administration (Ramakrishnan et al., 2021)	13.22 ± 2.38 (ERRα-luc-antagonist)
Camostat mesylate (59721–29–8)	Antiviral	Serine protease inhibitor	A phase I study conducted with high-dose of camostat shown to be safe thus providing a rationale for COVID-19 treatment (Kitagawa et al., 2021)	1.94 ± 0.66 (HDAC I/II antagonist)
Chloroquine (50–63–5)	Antimalarial	Lysosome inhibitor (Al-Bari, 2015)	A phase 2 randomized study demonstrated no reduction in the need for supplemental oxygen, invasive ventilation or death in severe COVID-19 patients (Galan et al., 2021)	30.10 ± 11.9 (AChE)
Chlorpromazine (50–53–3)	Antipsychotic and antiemetic	Post-synaptic blockade at dopamine (D2) receptor and antiemetic affect is by the combined blockade at D2, H1 (histamine), and M1 (muscarinic) receptors (Mann and Marwaha, 2020)	A pilot, randomized single blind therapeutic trial was proposed in COVID-19 patients who are requiring respiratory support without the ICU needs (Plaze et al., 2020)	29.82 ± 0.01 (TGFβ-bla-antagonist)
Colchicine (64–86–8)	Anti-inflammatory	Downregulates multiple inflammatory pathways (Leung et al., 2015)	A randomized, double-blinded, placebo-controlled clinical trial identified the colchicine use reduced the length of both supplemental oxygen use and hospitalization in moderate to severe COVID-19 patients (Lopes et al., 2021)	0.03 ± 0.01 (FXR-bla-antagonist)
				0.86 ± 0.20 (TGFβ-bla-antagonist)
				0.08 ± 0.01 (Shh-luc-antagonist)
Curcumin (458–37–7)	Anti-inflammatory	Attenuates inflammatory response of TNF-α stimulated endothelial cells (Alok et al., 2015)	The administration of nano-curcumin in COVID-19 patients has shown to be effective in controlling the inflammatory immune responses (Hassaniazad et al., 2021)	35.48 ± 0.01 (ARE-bla-agonist)
				12.20 ± 8.88 (Mitotox)
Dexamethansone (50–02–2)	Corticosteroid	Bind to GR to mediate for gene expression and inhibits phospholipase A2 activity	A RECOVERY trial resulted in reducing 28-days mortality in COVID-19 patients who are receiving respiratory support (Group et al., 2021)	0.01 ± 0.001 (GR-bla-agonist)
Emetine (483–18–1)	Anti-protozoal and anti-viral	Protein synthesis inhibitor and NF-kB inhibitor (Miller et al., 2010)	There is no clinical trial identified for emetine on COVID-19, but several *in vitro* studies have shown its potential as anti-SARS-CoV-2 agent by reducing ACE2 mRNA protein levels (Lee et al., 2021)	0.25 ± 0.01 (Aromatase)
				10.73 ± 2.25 (ERα-bla-antagonist)
				0.69 ± 0.05 (ERα-luc-antagonist)
				0.34 ± 0.04 (TGFβ-bla-antagonist)
				0.11 ± 0.02 (PXR-luc-agonist)
Febuxostat (144060–53–7)	Antigout	Xanthine oxidase inhibitor	A trial suggested that febuxostat may be considered as an alternative treatment to hydroxychloroquine for COVID-19 infection (Davoodi et al., 2020)	0.08 ± 0.02 (ERRα-luc-agonist)
				0.12 ± 0.06 (PGC/ERRα-luc-agonist)
Fluvoxamine (54739–18–3)	Antidepressant	Selective serotonin reuptake inhibitor (Sukhatme et al., 2021)	Prevented clinical deterioration in adult COVID-19 outpatients (Lenze et al., 2020)	11.45 ± 0.75 (AR-luc-agonist)
				28.23 ± 2.30 (PXR-luc-agonist)
Hydrocortisone (50–23–7)	Corticosteroid	GR agonist	Unable to provide the estimates of the beneficial effects compared with placebo due to the lower sample size from a randomized, placebo-controlled trial of adults with COVID-19 and severe hypoxia (Munch et al., 2021)	0.07 ± 0.02 (GR-bla-agonist)
Imatinib mesylate (220127–57–1)	Anticancer	Tyrosine-kinase inhibitor (BCR/ABL)	A randomized, placebo-controlled trial suggest that the observed effect on survival indicates that imatinib might confer clinical benefits in COVID-19 patients (Aman et al., 2021)	20.17 ± 1.31 (CAR-luc-agonist)
				18.74 ± 2.15 (PXR-luc-agonist)
Ivermectin (71827–03–7)	Anthelmintic	Angiotensin II receptor inhibitor	In a randomized clinical trial, ivermectin has not shown any significant improvement in treating mild COVID-19 patients (Lopez-Medina et al., 2021), but there was an observed trend in reducing the hospital stay for ivermectin-treated group (Abd-Elsalam et al., 2021)	1.18 ± 0.98 (FXR-bla-antagonist)
Lopinavir (192725–17–0)	Antiretroviral	HIV protease inhibitor	The clinical safety and effectiveness were evaluated using lopinavir/ritonavir with/without interferon-β-1a and hydroxychloroquine in COVID-19 patients and neither of them have shown significant improvement (Ader et al., 2021)	21.38 ± 9.84 (ARE-bla-agonist)
				27.93 ± 5.02 (AR-luc-antagonist)
				23.05 ± 1.50 (ERα-bla-antagonist)
				23.71 ± 0.01 (ERRα-luc-antagonist)
				32.73 ± 1.32 (Mitotox)
				18.29 ± 7.24 (PGC/ERRα-luc-antagonist)
				18.36 ± 9.98 (PXR-luc-agonist)
				22.18 ± 1.50 (TGFβ-bla-antagonist)
				16.02 ± 1.04 (PXR-luc-agonist)
Ritonavir (155213–67–5) Losartan (114798–26–4)	Antihypertensive	Angiotensin II receptor inhibitor (Timmermans et al., 1995)	A multi-center phase II randomized clinical trial of losartan on mild COVID-19 patients has shown no significant effect on viral load (Puskarich et al., 2021)	11.10 ± 2.50 (Shh-luc-antagonist)
Montelukast (151767–02–1)	Anti-inflammatory	Leukotriene receptor inhibitor	Resulted in fewer events of clinical deterioration among hospitalized COVID-19 patients (Khan et al., 2021)	24.08 ± 9.54 (Mitotox)
Nafamostat mesylate (82956–11–4)	Antiviral	Serine protease inhibitor	A randomized clinical trial evaluated the therapeutic effect and safety of nafamostat on COVID-19 pneumonia (Moon et al., 2021) and other study found no significant improvement in the treatment group (Zhuravel et al., 2021)	1.11 ± 0.25 (HDAC I/II antagonist)
Niclosamide (50–65–7)	Anthelmintic and anticestodal	Uncoupler	A randomized controlled open label clinical trial has shown niclosamide therapy to be clinically effective and relatively safety (Abdulamir et al., 2021)	0.48 ± 0.13 (CAR-luc-agonist)
				0.15 ± 0.03 (ERRα-luc-agonist)
				2.38 ± 0.16 (HDAC I/II antagonist)
				0.42 ± 0.20 (Mitotox)
	Antiviral			0.19 ± 0.04 (PGC/ERRα-luc-agonist)
				3.21 ± 2.33 (PXR-luc-agonist)
Nitazoxanide (55981–09–4)	Antiprotozoal and antiviral	Pyruvate: ferredoxin/flavodoxin oxidoreductase (PFOR) cycle inhibitor and viral replication suppressor	A randomized, placebo-controlled trial has shown early nitazoxanide therapy reduced viral load significantly (Rocco et al., 2021)	0.52 ± 0.06 (CAR-luc-agonist)
				0.53 ± 0.15 (ERRα-luc-agonist)
				4.80 ± 3.89 (GR-bla-agonist)
				1.60 ± 0.10 (HDAC I/II antagonist)
				9.15 ± 2.93 (Mitotox)
				1.11 ± 0.51 (PGC/ERRα-luc-agonist)
				15.30 ± 4.93 (PXR-luc-agonist)
Ribavirin (36791–04–5)	Antiviral	Inosine 5′-Monophosphate Dehydrogenase (IMPDH) Inhibitor (Te et al., 2007)	A trial concluded ribavirin in combination with nitazoxanide, ivermectin, and zinc supplement effectively cleared the SARS-CoV-2 from the nasopharynx in a shorter time in mild COVID-19 patients (Elalfy et al., 2021)	7.03 ± 5.45 (PGC/ERRα-luc-agonist)

*AC_50_ values of the compounds confirmed from the Tox21 follow-up assays, except for azithromycin, bromhexine, fluvoxamine, and lopinavir for which the values obtained from Tox21 screenings. The values are expressed as mean ± SD from three experiments, except for chloroquine, chlorpromazine, hydrocortisone, and montelukast for which the values are from two experiments.

### Tox21 qHTS Assay Panel

The qHTS data used in this study were generated from the Tox21 program, which is an inter-federal agency collaboration. The Tox21 program is mainly aimed at developing better toxicity assessment methods and its goal is to quickly and efficiently test whether certain chemicals have the potential to disrupt processes in the human body that leads to adverse effects (Tice et al., 2013). The Tox21 10K compound library consists of around 10,000 environmental chemicals to which humans are exposed on a routine basis through food or environment and approved drugs for clinical use that are structurally diverse (Richard et al., 2021). These compounds have been screened against various biological targets and signaling pathways in a battery of cell-based assays on a fully automated robotic system (Attene-Ramos et al., 2013). Several Tox21 screenings were performed in both agonist and antagonist modes for a particular assay to identify the inducers and inhibitors respectively. The Tox21 qHTS assay data from more than 75 assays testing for different biological endpoints are publicly available (https://tripod.nih.gov/tox21/assays/).

**FIGURE 1:**
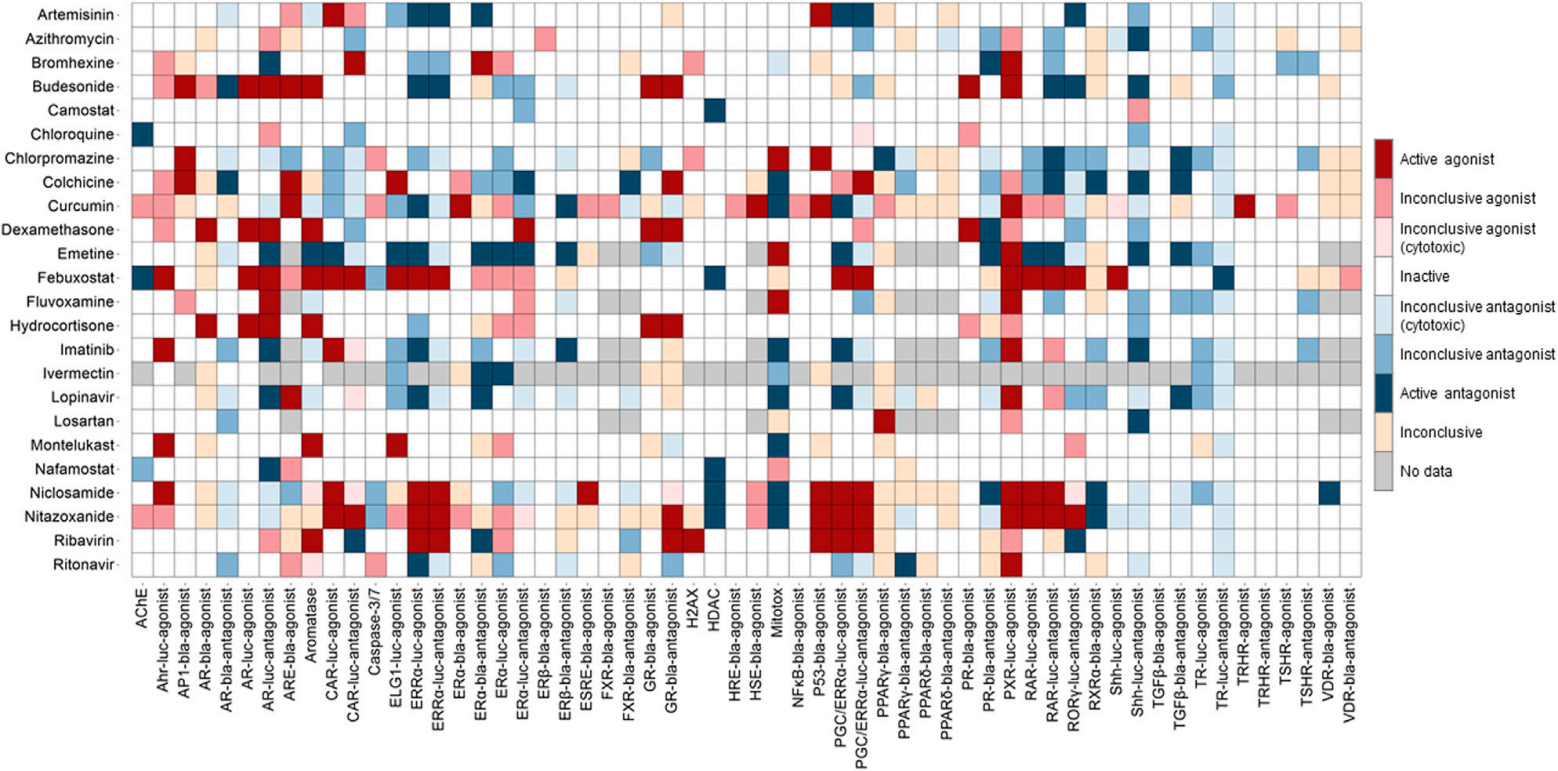
Activity of the drugs from Tox21 screenings. In the heat map, each column is an assay readout and each row is a drug. The heatmap is colored by the activity. The darkest of red and blue indicates the most active agonists and antagonists respectively. The other shades of red and blue indicates the respective inconclusives and in majority of the assays the drugs are inactive.

Concentration-response titration points for each compound from qHTS were fitted to a four-parameter Hill equation that yields concentration of half-maximal activity (AC_50_) and maximal response (efficacy) values (Huang et al., 2011). Classes 1–5 were assigned to the compounds based on the type of concentration-response curve observed. The activity outcomes (Figure 1 **)** for each compound from a particular assay were categorized according to the previously described criterion (Huang, 2016). Compounds that showed activation or inhibition were defined as agonists or antagonists respectively. Among agonists/antagonists, compounds were further defined as active agonists/antagonists with curve classes 1.1, or 2.1, and class 5 due to super potency; agonists/antagonists with curve classes 1.2, or 2.2; inconclusive agonist/antagonist with all other non-5 curve classes; inactives with curve class 4; inconclusive for which the activity direction cannot be determined. Inconclusive agonist/antagonists are further categorized based on the poor curve quality, and cytotoxicity of the compounds showing inhibitory phenotype.

**FIGURE 2:**
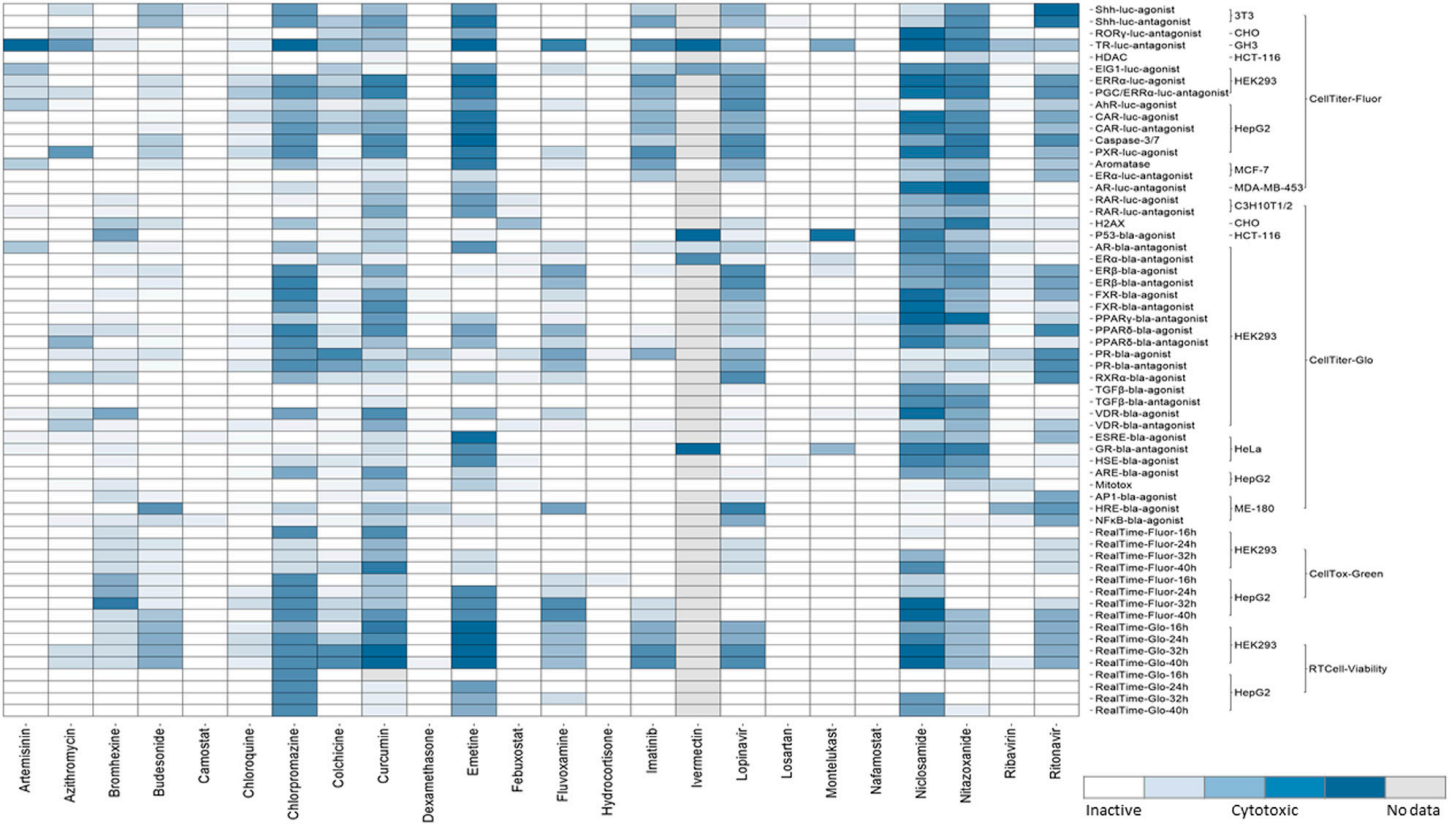
Cytotoxicity of the drugs from Tox21 screenings. In the heat map, each row is an assay readout and each column is a drug. The heatmap is colored by the activity. The different shades of blue indicates the cytotoxicity of the drugs in a particular assay. The cytotoxic assays performed for Tox21 screenings are grouped mainly into four categories- CellTiter-Fluor, CellTiter-Glo, CellTox-Green, and RT Cell-Viability and further subgroups are the cell types used for each assay.

When the perturbation of a cellular signaling pathway leads to adverse outcomes, this pathway can be considered a toxicity pathway. The Tox21 assay panel includes many cell-based assays that encompass multiple toxicity pathways. Many of these cell-based assays were reporter gene assays that measure β-lactamase (bla) or luciferase (luc) activities (Hsu et al., 2017). The cell lines used in Tox21 screens contain a reporter gene under control of the response elements for a specific target or signaling pathway that was stably integrated into different cell types, and wild-type cells were also used to test some targets. Tox21 screens were performed using immortalized and stably transfected cell lines because of their higher expression levels of defined molecular targets/cellular pathways and availability in a wide range of assay technologies. The background cell types of the Tox21 reporter gene assays that were used in the qHTS of chemicals include 3T3 (mouse embryonic fibroblasts), C3H10T1/2 (mouse fibroblasts), CHO (Chinese hamster ovary), GH3 (rat pituitary), HCT-116 (colorectal carcinoma), HEK293 (human embryonic kidney), HeLa (cervical carcinoma), HepG2 (hepatocellular carcinoma), ME-180 (cervical carcinoma), MCF-7 (breast carcinoma), and MDA-MB-453 (breast carcinoma). The assay panel also includes cell viability counter screen assays that were used to determine the cytotoxicity of a given compound, and majority of Tox21 assays with certain target/pathway have been performed in parallel with cytotoxicity assay in the same cell type as shown in Figure 2. Four viability assays (Promega Corporation, Madison, WI) with different readouts were implemented for Tox21 screenings (Figure 2). To determine the relative number of viable cells, fluorescence-based assays including CellTiter-Fluor™ (measures the protease activity in intact viable cells), and CellTox™ Green (measures the changes in membrane integrity of the cells in culture), and luminescence-based assays including CellTiter-Glo^®^ (quantitation of ATP in metabolically active cells), and RealTime-Glo™ MT Cell Viability (RT Cell Viability, measures the reducing potential of cells and thus metabolism) were used. CellTox™ Green and RT Cell Viability assays were used to measure cytotoxicity continually in the same well at different time intervals up to 40 h by reading fluorescence (Fluor) and luminescence (Glo) respectively in both HepG2 and HEK293 cells.

The qHTS data generated from this assay panel were used to evaluate the effect of several COVID-19 drug candidates on various molecular targets and signaling pathways. The *in vitro* assay activity and cytotoxicity profiles of the drug candidates from the Tox21 primary screens are shown as heat map illustrations in Figures 1,2 respectively. The AC_50_ values for the drug candidates tested against various targets/signaling pathways that were confirmed by our follow-up assays are given in Table 1. From these activity profiles, we have derived some insight on the mechanism of action for these drug candidates. The detailed findings will be discussed in the following sections.

### Nuclear Receptor Signaling

Nuclear receptors (NRs) are ligand-activated transcription factors that play an important role in cellular processes, including cell differentiation, proliferation, and metabolism. The Tox21 assay panel contains a number of assays that measure nuclear receptor (NR) signaling pathways (Huang et al., 2011), including androgen receptor (AR) (Lynch et al., 2017), aryl hydrocarbon receptor (AhR), estrogen receptor alpha, and beta (ERα, and ERβ) (Huang et al., 2014), estrogen-related receptor alpha (ERRα) (Lynch et al., 2018), ERRα paired with the peroxisome proliferator-activated receptor γ coactivator (PGC/ERRα), constitutive androstane receptor (CAR) (Lynch et al., 2019), farnesoid X receptor (FXR) (Hsu et al., 2014), glucocorticoid receptor (GR), peroxisome proliferator-activated receptors delta, and gamma (PPARδ, and PPARγ), pregnane X receptor (PXR) (Lynch et al., 2021), progesterone receptor (PR), retinoic X receptor alpha (RXRα), retinoid-related orphan receptor gamma (RORγ), thyroid hormone receptor (TR) (Paul-Friedman et al., 2019), and vitamin D receptor (VDR).

Several drugs currently under therapeutic trials for COVID-19 showed activities in the Tox21 NR assays (Figure 1). Drugs such as artemisinin, budesonide, colchicine, curcumin, dexamethasone, emetine, febuxostat, lopinavir, niclosamide, nitazoxanide, and ribavirin showed promiscuous activities in the NR signaling assays. Steroid hormone receptors, a subfamily of NRs including androgen, estrogen, progesterone, and glucocorticoid receptors in the lung, were reported for their role in regulating ACE2 and TMPRSS2 expression, which facilitates SARS-CoV-2 cell entry into host cell (Leach et al., 2021).

Estrogen was reported to regulate the expression of ACE2 (Stelzig et al., 2020). Recent study suggested that selective ER modulation, a potential therapeutic approach for attenuating the cytokine storm associated with COVID-19 (Calderone et al., 2020). Tox21 screenings include several assays testing the compounds activities against ERα, ERβ, and ERRα signaling pathways. Artemisinin, bromhexine, budesonide, emetine, febuxostat, niclosamide, nitazoxanide, and ribavirin were shown to be active modulators in our follow-up ER assays. Artemisinin and its derivatives are widely used as antimalarial agents that act by causing protein damage and inhibiting parasite proteasome function (Bridgford et al., 2018) and no serious side effects have been reported yet for the use of artemisinin (Meshnick, 2002). The plant extracts from Artemisia annua L, consisting of artemisinin derivatives have shown *in vitro* efficacy against SARS-CoV-2 and its variants (Nair et al., 2021; Nair et al., 2022). A trial with artemisinin-piperaquine treatment in patients with mild-to-moderate COVID-19 showed that this drug combination significantly shortened the infection time in the body (Li G. et al., 2021). Its ability to significantly improve symptoms in mild COVID-19 patients upon treatment with no severe side effects and its inhibitory effect on estrogen and related receptors suggest that artemisinin could have therapeutic values against SARS-CoV-2. Bromhexine, a mucolytic cough suppressant, was identified as a TMPRSS2 protease inhibitor from a chemical library screen for the identification of lead compounds to suppress prostate cancer metastasis (Lucas et al., 2014). According to the Tox21 primary screening results, bromhexine was active in several NR screening assays such as AR, CAR, ERα, PR, and PXR, and its activities yet to confirm through follow-up assays (Figure 1; Table 1). Emetine showed activities against multiple Tox21 assay targets, such as activation of PXR signaling, and inhibition of aromatase, ERα, and TGFβ signaling pathways. Aromatase is an enzyme that is responsible for estrogen synthesis and maintaining the normal balance of androgen and estrogen. We used an aromatase assay, which is a non-receptor mediated mechanism to identify aromatase inhibitors (Chen et al., 2015). *In vitro* studies have confirmed that emetine inhibits the SARS-CoV-2 replication (Choy et al., 2020; Kumar et al., 2021). Due to the toxic side effects associated with the use of emetine, lower doses of emetine could be a potentially effective anti-SARS-CoV-2 therapy (Bleasel and Peterson, 2020), as it was shown to block the virus entry into Vero cells (Wang et al., 2020). Though emetine has not been tested in clinical studies of COVID-19 yet, it has potential therapeutic value based on its ability to block viral entry *in vitro* and its activities against multiple Tox21 confirmation assays.

The inhibitors of AR pathway such as 5-alpha reductase inhibitors, were shown to reduce the levels of ACE2 and TMPRSS2, whereas activation of AR had the opposite effect of increasing the expression of viral receptor (Samuel et al., 2020). Glucocorticoids inhibit the interleukin (IL)-1 and IL-6 formation and stimulate the production of lipocortin that inhibits phospholipase A2 activity which decreases the formation of arachidonic acid from phospholipids, a precursor of prostanoids and leukotrienes (Amano et al., 1993). Hence these actions of glucocorticoids produce anti-inflammatory, and immunosuppressive properties. Recent studies support the use of glucocorticoids in the treatment of severe COVID-19 infections (Alexaki and Henneicke, 2021). The *in vitro* activities of GR activators such as dexamethasone and hydrocortisone against the Tox21 assays are shown in Figure 1, and these two compounds showed minimal cytotoxicity in the viability assays (Figure 2). The Tox21 library contains dexamethasone in various salt forms, including acetate, dipropionate, sodium phosphate, and valerate. In addition to GR activation, all these forms showed activation of AR, and PR in the Tox21 primary screens, but their activities are yet to be confirmed through follow-up assays.

FXR regulates bile acid synthesis, as well as lipid and glucose metabolism. The Tox21 10K compound collection was profiled for FXR modulators using a cell-based FXR-bla reporter gene assay (Hsu et al., 2014). Our secondary assays demonstrated that ivermectin and its analogs have antagonist activity against chenodeoxycholic acid-mediated FXR binding (Hsu et al., 2016a). Ivermectin was reported to inhibit the replication of SARS-CoV-2 *in vitro* (Caly et al., 2020). In a randomized clinical trial, ivermectin has not shown any significant improvement in treating mild COVID-19 patients (Lopez-Medina et al., 2021), but there was an observed trend in reducing the hospital stay for ivermectin-treated group (Abd-Elsalam et al., 2021). Its combination trials with doxycycline or nitazoxanide, ribavirin, and zinc were reported to help patients recover sooner from the disease (Mahmud et al., 2021) and shown to be effective in clearing the infection from the nasopharynx in shorter times (Elalfy et al., 2021) respectively. A recent study has identified that FXR participates in ACE2 expression in multiple tissues that are affected by COVID-19, and demonstrated that approved FXR inhibitors including z-guggulsterone and ursodeoxycholic acid downregulate ACE2 expression and reduced susceptibility to SARS-CoV-2 infection in lung, cholangiocyte and gut organoids (Brevini et al., 2021).

### Toxicity Related Signaling Pathways and Targets.

The Tox21 screening assay panel also constitutes assays that measure chemical activities in several stress related pathways, including activator protein 1 (AP1) (Zhu et al., 2021), p53 (Ooka et al., 2022), enhanced level of genome instability gene 1 (ElG1) (Fox et al., 2012), caspase-3/7, antioxidant response element (Nrf2/ARE) (Zhao et al., 2016), heat shock response element (HSE) (Hancock et al., 2009), endoplasmic reticulum stress response element (ESRE) (Bi et al., 2015), nuclear factor-kappa B (NFkB) (Miller et al., 2010), histone variant H2AX, hypoxia response element (HRE) (Xia et al., 2009), and mitochondrial toxicity (mitotox) (Attene-Ramos et al., 2015); developmental related cellular pathways, such as the transforming growth factor beta signaling (TGF-β, SMAD-dependent) (Wei et al., 2019), retinoic acid response element (RAR, retinol signaling pathway) (Chen et al., 2016), and sonic hedgehog pathway (Shh/Gli1); GPCR/cAMP (thyroid stimulating hormone receptor, TSHR), and GPCR/calcium signaling (thyrotropin-releasing hormone receptor, TRHR) pathways; and other targets such as acetyl cholinesterase (AChE) (Li et al., 2021c), aromatase (Chen et al., 2015), and histone deacetylases I/II (HDAC I/II) (Hsu et al., 2016b).

Mitochondrial membrane potential (MMP) is a key parameter for assessing mitochondrial function. In addition to their major role in metabolic pathways, mitochondria were also known to mediate antiviral immunity through mitochondrial antiviral signaling proteins (MAVS) present in their outer membrane (Mohanty et al., 2019). One of the Tox21 assays uses a mitochondrial membrane potential indicator (m-MPI) to quantify the changes in MMP in HepG2 cells (Attene-Ramos et al., 2015). Some of the current drug candidates in COVID-19 clinical trials, including curcumin, montelukast, niclosamide, and nitazoxanide, affected mitochondrial function by decreasing the membrane potential according to our study (Table 1). Curcumin has been administered in mild hospitalized COVID-19 patients in the nano-form to investigate its use as a complementary therapeutic agent due to its anti-inflammatory effect. The nano-curcumin therapy was able to efficiently modulate the inflammatory state in COVID-19 patients. The results showed that the treatment decreased the expression of IL-1β and IL-6 mRNA but was not able to diminish the mRNA levels of IL-18 and TNF-α, and aided in the overall recovery of the patients (Valizadeh et al., 2020). However, curcumin and its analogues appear to be challenging due to the lack of chemical stability, solubility, selective target activity and bioavailability and had been classified as a pan-assay interference compound (PAIN), and therefore it is an improbable lead (Nelson et al., 2017). Our secondary follow-up study showed curcumin as an active agonist in Nrf2/ARE signaling pathway assay. Oxidative stress plays a role in the pathogenesis of a variety of diseases ranging from cancer to neurodegeneration. Oxidative stress response is coordinated at the transcriptional level by nuclear factor erythroid 2-related factor 2 (Nrf2) (Kensler et al., 2007). A cell based ARE-bla reporter gene assay was used to identify the compounds that modulate the Nrf2/ARE signaling pathway (Zhao et al., 2016). A recent study demonstrated that expression of Nrf2-driven genes are suppressed in biopsies of COVID-19 patients by the treatment of cells with Nrf2 agonists, and these agonists were shown to be effective in limiting viral replication and suppressing host inflammatory responses of several human pathogenic viruses, including SARS-CoV-2 (Olagnier et al., 2020).

The disruption of transforming growth factor beta (TGFβ) signaling during embryonic development can affect morphogenesis and differentiation through SMAD dependent or independent pathways. For Tox21 screening, we used an assay to identify potential disruptors of the SMAD-dependent TGFβ signaling pathway (Wei et al., 2019). SARS-CoV-2 triggers a TGFβ instructed chronic immune reaction in severe COVID-19 patients (Ferreira-Gomes et al., 2021). Our follow-up assays showed chlorpromazine, and colchicine as TGFβ signaling pathway inhibitors. Chlorpromazine is a phenothiazine and these phenothiazine derivatives possess anti-cancer, -viral, -bacterial and -fungal activities (Otreba et al., 2020). Based on the Tox21 screening results, chlorpromazine is one of the few drugs that exerted cytotoxicity against most of the cell types (Figure 2). This drug was reported to induce non-neurological side effects including dizziness, blurred vision, angle-closure glaucoma in elder patients, and may cause sedation due to the blockade of H1 receptors (Mann and Marwaha, 2020). This drug has shown *in vitro* inhibition against SARS-CoV-2 replication, and *in vivo* studies have shown that the drug protected mice from severe clinical disease of SARS-CoV (Weston et al., 2020). Colchicine showed inhibition in FXR, TGFβ, and Shh signaling pathways from our studies. The mechanism of action of colchicine is tubulin disruption and its anti-inflammatory and anti-fibrotic activities aid for its therapeutic ability (Leung et al., 2015).

Another essential pathway for normal embryonic development is the Shh signaling (Ingham and McMahon, 2001) and the dysregulation of which can lead to severe developmental defects. Our primary screenings showed azithromycin as active inhibitors of Shh signaling pathway, and its activity yet to be confirmed through follow-up assays. Azithromycin with reported antiviral activity (Gielen et al., 2010) has been in current treatment trials for COVID-19, which is given alone (Group, 2021) or in combination with hydroxychloroquine (Cavalcanti et al., 2020) to evaluate its potential antiviral effect, however the clinical outcomes confirmed no improvement compared with standard care. The adverse effects of azithromycin were considered rare, but there is possible cardiac risk associated with its use (Lu et al., 2015). The Shh and Wnt signaling pathways were hypothesized to play a role in pneumomediastinum-related tracheal lesions in COVID-19 patients (Baratella et al., 2021). Pneumomediastinum is an uncommon condition in which air leaks into the mediastinum and an increased incidence of pneumomediastinum was reported during the COVID-19 pandemic (Mart et al., 2021).

Endoplasmic reticulum plays a role in cellular signaling and stress response, and its dysregulation has been associated with several diseases. A Tox21 assay was developed to identify the endoplasmic reticulum stress inducers (Bi et al., 2015). Niclosamide showed to be an ESRE inducer from our primary screening, yet to confirm its activity through follow-up assay. It has been suggested that sigma-1 receptor in endoplasmic reticulum plays a key role in replication of SARS-CoV-2 in the host cells and the subsequent endoplasmic reticulum stress due to the viral replication may contribute to the cytokine storm (Hashimoto, 2021). Fluvoxamine, an antidepressant and a selective serotonin reuptake inhibitor, has a strong affinity for the sigma-1 receptor that functions as an endoplasmic reticulum molecular chaperone (Sukhatme et al., 2021). A randomized trial reported that fluvoxamine could prevent clinical deterioration in COVID-19 outpatients after the treatment, but the sample size was relatively small in the study (Lenze et al., 2020).

Cholinesterases, acetylcholinesterase (AChE) and butyrylcholinesterase (BChE), are involved in neurotransmission termination by hydrolyzing the choline-based esters (Pohanka, 2011). A study demonstrated that the cholinesterase levels in serum were associated with the severity and mortality in COVID-19 pneumonia patients (Nakajima et al., 2021). The Tox21 screens included assays to identify inhibitors of AChE (Li et al., 2021c) and BChE (Li et al., 2021b), which have therapeutic potential for diseases such as Alzheimer’s. Chloroquine displayed inhibitory effect on AChE in our confirmation assays. Chloroquine and its analog hydroxychloroquine, collectively called as 4-aminoquinolines, have been used as anti-malarial agents. 4-Aminoquinolines inhibit the autophagy machinery through lysosomal inhibition, and because of their lysosomal inhibitory action (Al-Bari, 2015), these drugs have therapeutic applications in oncology. Cardiac complications and retinal toxicity are associated with chloroquine and hydroxychloroquine, which need to be considered when applying these drugs for the treatment of viral infections (Chatre et al., 2018; Ruamviboonsuk et al., 2020). Chloroquine/hydroxychloroquine showed significant antiviral effect against SARS-CoV-2 *in vitro* by inhibiting the terminal phosphorylation of ACE II (Shukla et al., 2020). One of the recent published studies which included the randomized controlled trial for evaluating the efficacy of chloroquine/hydroxychloroquine in severe COVID-19 patients reported significant worsening of the disease condition leading to the need for mechanical ventilation (Rea-Neto et al., 2021).

The histone deacetylases I/II (HDACs) are a group of epigenetic enzymes that regulate histone deacetylation. HDAC is a therapeutic target because of its capability of introducing epigenetic modifications that contribute to the onset and progression of several human diseases (Berdasco and Esteller, 2013). Clinically approved HDAC inhibitors were shown to be effective in preventing the entry of SARS-CoV-2 into the host cell by suppressing the ACE2 receptor (Liu et al., 2020; Takahashi et al., 2021). Tox21 primary screens and follow-up confirmation assays identified camostat mesylate, febuxostat, nafamostat, niclosamide, and nitazoxanide as potential HDAC inhibitors (Hsu et al., 2016b). TMPRSS2 inhibitors such as camostat and nafamostat had inhibitory effect on HDAC from our study, which can suggest that TMPRSS2 inhibitors can also be putative HDAC inhibitors.

## Summary

This study reviewed the drugs that are under current clinical trials as potential COVID-19 therapeutics and evaluated their activities across various targets and pathways tested in Tox21 qHTS assays. Multiple cellular signaling pathways get recruited upon the viral entry into the host cells to facilitate viral replication, inflammation, acute lung injury and drugs that effect these signaling pathways could have potential therapeutic effects. The drugs from our study, which are employed in the current therapeutic trials of COVID-19 that have shown beneficial effect by reducing viral loads include artemisinin, bromhexine, budesonide, curcumin, fluvoxamine, and nitazoxanide and the drugs that have shown significant improvement from the severity of disease include colchicine, imatinib, and niclosamide. Most of these drugs were shown to be active modulators in our nuclear receptor signaling pathway assays. Several signaling pathways including Nrf2/ARE, endoplasmic reticulum stress, and targets like HDAC were reported for their role in regulating ACE2 and TMPRSS2 expression, which are the main host cell factors that aid in SARS-CoV-2 pathogenicity. Drugs targeting such signaling pathways could have therapeutic potential for COVID-19 and considering the cytotoxicity data obtained from *in vitro* assays can play a vital role in the drug repurposing studies for COVID-19 therapeutics as well. Some of the drugs in our study were shown to be cytotoxic against a wide range they include chlorpromazine, curcumin, emetine, lopinavir, ritonavir, niclosamide, and nitazoxanide. The cytotoxicity of these compounds might be due to their target promiscuity as shown by their activities in multiple Tox21 *in vitro* assays. Although *in vitro* cytotoxicity assays were used to screen chemicals/drugs for their relative toxicities at micro-molar concentrations, these assays can identify a potential hazard due to the multiple uses or high doses of the drugs when administered in humans. These qHTS data are publicly available and can provide valuable information on drug activities and their off-target effects, which can be further investigated for their potential uses in treating COVID-19 infection.
